# Safety evaluation of a β-mannanase enzyme preparation produced with *Thermothelomyces thermophilus* expressing a protein-engineered β-mannanase gene

**DOI:** 10.1371/journal.pone.0243647

**Published:** 2020-12-10

**Authors:** Andreas Kern, Diane Shanahan, Roland Buesen, Dominik Geiger

**Affiliations:** 1 BASF Corporation, San Diego, California, United States of America; 2 Experimental Toxicology and Ecology, BASF SE, Ludwigshafen am Rhein, Germany; 3 Global Service Cluster Safety, BASF SE, Ludwigshafen am Rhein, Germany; Universidade Federal de Minas Gerais, BRAZIL

## Abstract

Mannanase 19287 enzyme is an engineered β-mannanase that can be added to diets for animals raised for human consumption to hydrolyze β-mannans. Established toxicological analyses were conducted with the enzyme preparation to ensure the safety of this product for the intended use. The mannanase 19287 preparation was produced with *Thermothelomyces thermophilus* strain DSM 33149. *In vitro* toxicity studies presented here used dosages of the mannanase 19287 test articles up to 5000 μg/plate. For *in vivo* toxicity studies in Wistar rats, test articles were administered at 5.1 mg/L for inhalation toxicity and up to 15,000 mg/kg rat feed for oral toxicity, based on the Total Organic Solids (TOS) content in each test article. No treatment related adverse effects were reported in any study. The No Observed Adverse Effect Levels in the high dose group of the subchronic oral toxicity study were calculated as 1117–1298 mg TOS/kg bw/day in rats. Comparing these values to an Estimated Daily Intake for poultry demonstrated safety factors larger than 5000. Our results confirm that *T*. *thermophilus* fulfills the recognized safety criteria for the manufacture of food enzyme preparations and represent the first peer-reviewed safety evaluation of an enzyme preparation by *T*. *thermophilus*. The results of the toxicity studies presented herein attest to the safety of the mannanase 19287 enzyme for its intended use.

## Introduction

Mannanases are enzymes capable of hydrolyzing mannans as either exo- (cleaving the terminal saccharides) or endo- (cleaving randomly within the polysaccharide chain) glucanases [1]. Endo-1,4-β-mannanases (E.C. 3.2.1.78) [2] are a class of glycosyl hydrolases that catalyze the random hydrolysis of (1→4)-β-D-mannosidic linkages in mannans, and galactomannans. Endo-1,4-β-mannanases are produced by a variety of organisms, including prokaryotes such as *Bacillus subtilis* and *Paenibacillus lentus* (formerly named *Bacillus lentus*), as well as eukaryotes, including fungi, such as *Aspergillus niger* and *Trichoderma reesei* [3].

Mannans are polysaccharides consisting of linear or branched chains of β-D-mannose with saccharide side chains consisting of mannose, galactose and glucose with additional acetylation of the saccharides; they are part of the soluble non-starch polysaccharide (SNSP) components of hemicellulose in plant fibers [4]. Mannans, galactans and other SNSPs affect the viscosity of aqueous solutions through their ability to tightly bind water. Viscous digesta in farm animals have negative effects on the digestion of feed and availability of nutrients to support the growth of poultry, swine and other animals for human consumption [5–7]. In particular, non-ruminants / monogastric animals like pigs and chickens do not generate sufficient cellulolytic enzymes to reduce viscosity in their digestive tracts [1, 8–10].

The mannanase 19287 enzyme is a member of the GH5 family of glycohydrolases (CAZy; http://www.cazy.org, [11, 12]) and is expressed in *Thermothelomyces thermophilus*, a filamentous fungus belonging to the order Sordariales in the phylum Ascomycota. *T*. *thermophilus* has previously been classified as *Myceliophthora thermophila* [13] and is a thermophilic fungus with optimal growth between 35°C and 48°C; it is known for rapid degradation of plant cellulosic substances [14]. Several lignocellulolytic enzymes have been identified in the genome, and several endogenous enzymes have demonstrated high thermostability and enzyme optima up to 69°C [15]. The wild-type *T*. *thermophilus* strain C1 was isolated first from soil in eastern Russia in 1990 and deposited in the Russian cell bank (accession number VKM F-3500-D). The C1 strain has been developed into versatile host- and production strains for industrial manufacture of enzymes [16].

The enzyme preparation is intended for use in animal feed to affect digesta viscosity and catalyze the degradation of mannan carbohydrate chains of hemicellulose under conditions found in the stomach of target animals. Digestion of the mannan carbohydrate components of hemicellulose may result in the reduction of gut and excreta viscosities and thereby increase the digestibility of grain food sources [5, 6].

In the present study, we report several toxicological tests that were conducted to evaluate the safety of the mannanase 19287 preparation for use as an animal feed ingredient. Specifically, the enzyme preparation was studied in a bacterial reverse mutation assay, an *in vitro* human lymphocytes micronucleus test, an acute inhalation toxicity study and a 90-day subchronic oral toxicity study in rats. In addition, *in vitro* skin and eye irritation studies were performed.

## Materials and methods

### Production strain generation

**Fig 1:**
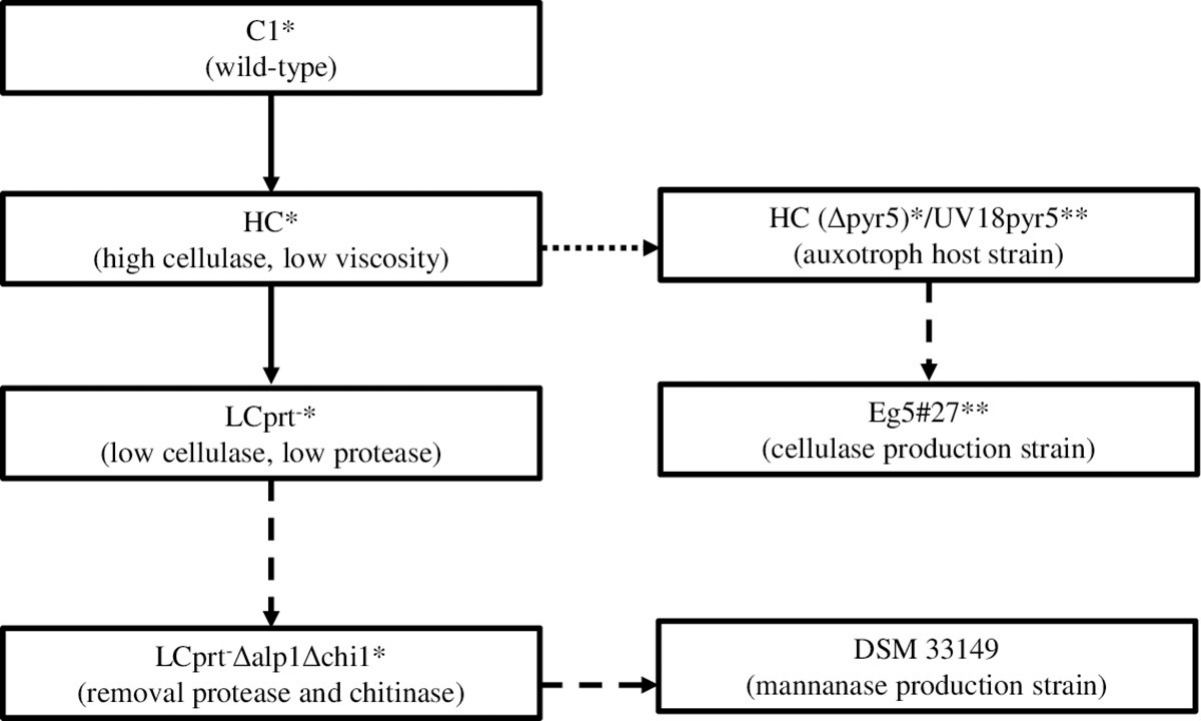
Strain lineage depiction of two *T*. *thermophilus* enzyme production strains. Eg5#27 (cellulase) and DSM 33149 (mannanase 19287, the subject of this publication) were derived from the C1 isolate and its descendant strains, using random mutagenesis, recombinant DNA techniques or further purification of HC strains. The strain nomenclature follows the Visser publication (*, [16]) and GRAS Notification 292 (**, [17]).

The mannanase 19287 production strain DSM 33149 is derived from the wild-type C1 strain of *T*. *thermophilus* that has been isolated and developed into an industrial-scale protein production system [16]. The C1 isolate was subjected to standard strain improvement approaches, including random mutagenesis by exposure to UV light or the chemical agent N-methyl-N’-nitro-N-nitrosoguanidine (NTG). Isolates were screened for increased enzyme production and advantageous changes in morphology. These approaches resulted in an optimized strain called HC [16] that showed reduced fermentation culture viscosity and a high level of cellulase secretion. Further development processes applied to the HC strain yielded novel strains that showed significant reductions in cellulase and protease activities, while maintaining the ability to secrete large quantities of recombinant proteins. Fig 1 shows a high-level overview of the strain development process from the wild-type C1 strain via the HC strain to subsequent enzyme production strains using the published strain nomenclature [16].

A cellulase preparation produced with *T*. *thermophilus* strain Eg5#27, a direct descendant of the HC strain, underwent standard toxicological studies and the manufacturer Dyadic International (USA), Inc., determined that the cellulase preparation is Generally Recognized as Safe (GRAS) for its intended use during beer, wine and juice production. Summaries of the studies were included in GRAS Notification GRN 292 submitted to the U.S. FDA. This notification and FDA’s “No questions” response letter are publicly available [17, 18]. To date, no peer-reviewed safety evaluations have been published for enzyme preparations produced in the *T*. *thermophilus* species.

The *T*. *thermophilus* HC strain is considered non-pathogenic and non-toxigenic based on the results of the direct descendant strain Eg5#27. The HC strain is also the parental strain of the *T*. *thermophilus* host strain LCprt^-^Δalp1Δchi1Δpyr5 (nomenclature per [16]) and the mannanase 19287 production strain *T*. *thermophilus* DSM 33149 (deposited at the German Leibniz Institute DSMZ-German Collection of Microorganisms and Cell Cultures). The above-mentioned publication [16] describes the strain lineage leading to the host strain LCprt^-^Δalp1Δchi1Δpyr5 (see Fig 1 for an overview). To create the host strain, the pyr-5 gene, coding for an orotate-phosphoribosyl-transferase enzyme, was inactivated, rendering the resulting host strain auxotroph for uridine.

The protein-engineered β-mannanase protein sequence was generated using modern biotechnology methods [19]; the sequence shows high homology with fungal mannanases belonging to the GH 5 family of the Carbohydrate-Active Enzymes database (CAZy; http://www.cazy.org [11, 12]). The protein-engineered modifications resulted in mannanase 19287, an enzyme that retained high activity under conditions found in the stomach of the target animal. In addition, the modifications also increased the temperature maximum of mannanase activity and the thermostability of the enzyme. The latter two characteristics allow for the retention of enzyme activity in animal feed following the high-temperature pelleting process common in the modern feed industry.

The genetic modifications of the *T*. *thermophilus* host strain, resulting in production strain DSM 33149 included transformations with a linearized mannanase expression cassette. Changes included the endogenous pyr5 gene to restore uridine autotrophy, and the amdS gene from *A*. *nidulans* to allow for the selection of transformants on plates containing acetamide as the only nitrogen source [20]. The linear expression cassette contained *T*. *thermophilus* genetic elements to ensure expression and proper targeting of the protein-engineered mature full-length endo-1,4-β-mannanase sequence. No plasmid vector-related DNA remained in the expression cassette. All transformations during the development of production strain DSM 33149 were performed on isolated protoplasts [21]. The procedure resulted in the generation of the production strain DSM 33149, showing expression of high levels of mannanase 19287 in the culture supernatant.

### Test article preparation

Three mannanase 19287 test articles for toxicological analyses were produced in a pilot plant with *T*. *thermophilus* production strain DSM 33149: (1) liquid concentrate Registry Batch (RB) 2 (subsequently referred to as RB2), (2) liquid concentrate RB5, and (3) spray-dried powder (SD). The pilot plant fermentation and recovery steps were representative of the commercial-scale manufacturing process for the enzyme preparations, using well-established industrial procedures [22, 23], and in accordance with current good manufacturing practices (cGMP) for animal food.

For production of test articles RB2 and RB5, the fermentation and recovery steps used in the manufacturing process were scaled down to equivalent unit operations in the pilot plant. After termination of the fermentation process by cell-kill, standard cake-, polish- and germ filtration steps were used to separate the non-viable production strain from the enzyme in the fermentation supernatant and clarify the enzyme solution [22, 23]. The enzyme solution was then concentrated using ultrafiltration and subjected to a final filtration step to obtain the liquid enzyme concentrate prior to storage. The liquid concentrate RB5 was also further processed via spray-drying, resulting in test article SD. The test articles were stored refrigerated (5°C). The Sponsor supplied the test articles to the study sites.

### Test article chemical and microbiological characterization

Prior to use in toxicological studies, test articles RB2, RB5 and SD were analyzed for their composition, including water content and residual ash to derive Total Organic Solids (TOS) content. The endo-1,4,-β-mannanase activity was determined using a validated enzyme assay.

In addition, RB2 and RB5 were tested for compliance with the microbial and chemical specifications established for enzyme preparations used in food (and feed) processing, as published in the Food Chemical Codex (FCC), Eleventh Edition, 2018 [24], and by the Joint FAO/WHO Expert Committee on Food Additives [25]. These specifications include the absence of antimicrobial activity, absence of *Salmonella* and *E*. *coli*, as well as limits on coliforms and lead. Lastly, the test articles RB2, RB5 and SD were tested for the potential presence of mycotoxins (Aflatoxin B1, Deoxynivalenol, Zearalenone, HT2-Toxin, T2-Toxin, Ochratoxin A, Fumonisin (FB1+FB2), and Sterigmatocystin).

### Bacterial reverse mutation assay

The bacterial reverse mutation assay was conducted in compliance with OECD Guideline 471, updated and adopted 21 July 1997, EU Commission Regulation 440/2008 (2008) and EPA OPPTS 870.5100 (1998). The study was performed in accordance with OECD Principles of Good Laboratory Practice (ENV/MC/CHEM(98)17). These principles are compatible with GLP regulations in the EU, the US (FDA and EPA) and Japan.

The vehicle used to deliver endo-1,4-β-mannanase test article RB5 to the test system was deionized water. Test article dilutions were prepared fresh before treatment and used within two hours of preparation.

The tester strains used were the *Salmonella typhimurium* histidine auxotrophs TA98, TA100, TA1535, and TA1537 [26], and *Escherichia coli* WP2 *uvr*A [27]. The bacterial strains TA 1535, TA 1537, TA 98, TA 100, and WP2 uvrA were obtained from Trinova Biochem GmbH (35394 Gießen, Germany).

Tester strains TA98 and TA1537 are reverted from histidine dependence (auxotrophy) to histidine independence (prototrophy) by frameshift mutagens. Tester strain TA1535 is reverted by mutagens that cause base pair substitutions. Tester strain TA100 is reverted by mutagens that cause both frameshift and base pair substitution mutations [26]. Specificity of the reversion mechanism in *E*. *coli* is sensitive to base pair substitution mutations, rather than frameshift mutations [27].

The thawed bacterial suspension was transferred into 250 mL Erlenmeyer flasks containing 50 mL nutrient medium. This nutrient medium contained nutrient broth (8 g/L) and NaCl (5 g/L). A solution of 50 μL ampicillin (25 μg/mL) was added to the strains TA 98 and TA 100.

The bacterial cultures were incubated in a shaking water bath for 4 hours at 37°C. The optical density of the culture broth was determined by absorption measurement and the obtained values indicated that the bacteria were harvested at the late exponential or early stationary phase (10^8^−10^9^ cells/mL).

Phenobarbital/β-naphthoflavone induced rat liver S9 was used as the metabolic activation system. The S9 was prepared and stored according to the currently valid version of the Envigo CRS GmbH SOP for rat liver S9 preparation. Each batch of S9 was routinely tested for its capability to activate the known mutagens benzo[a]pyrene and 2-aminoanthracene in the Ames test.

In Experiment I (the plate incorporation test), aliquots of *S*. *typhimurium* and *E*. *coli* strains were plated in the presence of 3, 10, 33, 100, 333, 1000, 2000, and 5000 μg endo-1,4-β-mannanase, solvent (deionized water, DMSO) or positive control substances (sodium azide [NaN3], 4-nitro-o-phenylene-diamine [4-NOPD], methyl methane sulfonate[MMS], or 2-aminoanthracene [2-AA], in metabolic activation samples), respectively. The stated concentrations account for the Total Organic Solids (TOS) content of 23.1% w/w in the test article RB5, using a correction factor of 4.33. All tests were performed in the presence of S9 mix or S9 substitution buffer.

In Experiment II (the pre-incubation test), *S*. *typhimurium* and *E*. *coli* strains were incubated each in the presence of 33, 100, 333, 1000, 2000, and 5000 μg endo-1,4-β-mannanase (concentrations corrected to account for TOS content of test article RB5), solvent or mutagen solution, respectively. Again, all tests were performed in the presence of S9 mix or S9 substitution buffer. After incubation, overlay agar was added to the mixes and the solutions were plated on the selective agar plates.

### *In vitro* micronucleus test in Human Peripheral Blood Lymphocytes (HPBL)

The *in vitro* micronucleus test in human lymphocytes was conducted in compliance with OECD Guideline 487, updated and adopted 29 July 2016, and with EU Commission Regulation 217/735 (2017). The study was performed in accordance with OECD Principles of Good Laboratory Practice (ENV/MC/CHEM(98)17). These principles are compatible with GLP regulations in the EU, the US (FDA and EPA) and Japan.

The vehicle used to deliver RB5 to the test system was deionized water. Test article dilutions were prepared immediately before use and used within two hours of preparation.

The study was performed at an accredited Contract Research Organization, ICCR-Roßdorf GmbH, Roßdorf, Germany and was approved by the test facility management on 14 August 2018. The study was assigned to study No. 1908004. The HPBL were obtained from two healthy, non-smoking individuals. All blood donors used in this study were of legal age. For every single blood donation, the blood donors gave their informed consent to use the blood cultures for scientific purposes. The consent was confirmed by personal signature. The lymphocytes from each donor had previously shown to respond well to proliferation. All donors had previously established low incidence of micronuclei in their peripheral blood lymphocytes.

The HPBL were cultured in a mix of 11% whole blood and DMEM/Ham’s F12 complete medium containing 10% fetal bovine serum (FBS), 200 mM GlutaMAX™, 100 units penicillin/mL, 100 μg/mL streptomycin, the mitogen phytohemeagglutinine (PHA; 3 μg/mL), 10 mM HEPES and heparin (125 U.S.P.-U/mL). The cultures were incubated under standard conditions (37 ±1°C in a humidified atmosphere of 5 ±1% CO_2_ in air) for 44–48 hours.

Mitomycin C (MMC, 1 μg/mL), Demecolcine (100 ng/mL) and Cyclophosphamide (CPA, 17.5 μg/mL) were dissolved and diluted in deionized water for use as positive control substances. Phenobarbital/β-naphthoflavone induced rat liver S9 was used as the metabolic activation system.

### Ninety-day repeated dose oral toxicity study in rats

The ninety-day oral toxicity study in rats was conducted at BASF Experimental Toxicology and Ecology Laboratories in Ludwigshafen, Germany, an AAALAC (Association for Assessment and Accreditation of Laboratory Animal Care)-approved laboratory. The study was assigned to BASF project No. 50C0147/18S047. The study was performed in accordance with the German Animal Welfare Act as well as the effective European Council Directive 2010/63/EU on the protection of animals used for scientific purposes, including all efforts to minimize suffering. This study was approved by the local authorizing agency for animal experiments (Landesuntersuchungsamt Rheinland-Pfalz, Koblenz, Germany) on 17 January 2019, as referenced by the approval number 23 177-07/G 17-3-063. The study was performed in compliance with OECD Guideline 408, updated and adopted 25 June 2018, EU Commission Regulation 440/2008 (2008), and US EPA Health Effects Guideline OPPTS 870.3100 (1998) and in accordance with OECD Principles of Good Laboratory Practice (ENV/MC/CHEM(98)17). These principles are compatible with GLP regulations in the EU, the US (FDA and EPA) and Japan.

The test article RB2 was supplied as a liquid concentrate and was mixed into the standard rat feed. Stability of the test-substance preparations in rat feed was confirmed for up to 35 days by enzyme activity measurements. The final activity of the mannanase 19287 in rat feed was confirmed using a validated enzyme activity test. The test-substance preparations were mixed weekly and stored at room temperature.

Eighty experimentally naïve Wistar rats (40 males and 40 females), 7 weeks old and a mean weight of about 147 grams (males) and 119 grams (females) at the start of the administration period, were obtained from Charles River Laboratories, Research Models and Services, Germany GmbH (97633 Sulzfeld, Germany). The animals were assigned to four treatment groups: Group 1, Control (0 ppm test article); Group 2, 6500 ppm test article; Group 3, 21700 ppm test article; and Group 4, 65000 ppm test article. Each treatment group included 10 animals each of each gender, respectively.

The test article was administered daily via the diet over a period of three months. Mortality/morbidity observations were performed twice daily (Mon–Fri) and at least once on weekends and holidays. If moribund animals were found, they would be sacrificed humanely and necropsied. Procedures addressing recognition and handling of moribund animals for this study type are defined in a standard operation procedure of the test facility (SOP-CUS 7.1.06). Briefly, upon observation of any of the following signs or conditions, the study director and/or the veterinarian would have been notified to decide on the appropriate action to alleviate pain or distress, including the humane sacrifice of the animal: prolonged inability to eat or drink; indications of severe pain, distress or suffering; rapid or continuing weight loss; abnormal appearance or activities; severe respiratory distress; bleeding, anemia, cyanosis; or palpable abdominal masses.

Animals were examined daily for abnormal clinical signs. Detailed clinical observations in an open field were performed in all animals prior to the administration period and thereafter at weekly intervals.

Body weight was determined before the start of the administration period in order to randomize the animals. During the administration period the body weight was determined on day 0 (start of the administration period) and thereafter at weekly intervals. Food consumption was recorded weekly. The mean daily intake of test article (group means) was calculated based upon individual values for body weight and mean food consumption per animal.

Ophthalmological examinations were performed before the beginning and towards the end of the administration period. Beside this, a functional observational battery as well as measurement of motor activity were carried out towards the end of the administration period.

Clinico-chemical and hematological examinations as well as urinalyses were performed towards the end of the administration period. Animals were anesthetized using isoflurane prior to blood sampling.

After the administration period, all animals were anesthetized using isoflurane, sacrificed by decapitation and assessed by gross pathology. Organ weights were determined followed by histopathological examinations of selected organs and tissues from the control and high-dose group animals.

### Acute inhalation toxicity in rats

The 4-hour dust exposure study to determine the acute inhalation toxicity of test article SD in male and female Wistar rats was conducted at BASF Experimental Toxicology and Ecology Laboratories in Ludwigshafen, Germany, an AAALAC-approved laboratory. The study was assigned to BASF project No. 13I0148/18I011 and was performed in accordance with the German Animal Welfare Act as well as the effective European Council Directive, including all efforts to minimize suffering. This study was approved by the local authorizing agency for animal experiments (Landesuntersuchungsamt Rheinland-Pfalz, Koblenz, Germany) on 21 June 2018, as referenced by the approval number 23 177-07/G 17-3-063. The study was conducted in compliance with OECD Guideline 403, updated and adopted 7 September 2009, EU Commission Regulation 260/2014 (2014), and US EPA Health Effects Guideline OPPTS 870.3100 (1998), respectively. The study was performed in accordance with OECD Principles of Good Laboratory Practice (ENV/MC/CHEM(98)17). These principles are compatible with GLP regulations in the EU, the US (FDA and EPA) and Japan.

The test was run with an actual measured concentration of 5.088 mg test article SD/L (analytical concentration, limit test). The test article SD was disaggregated in a mixer and a dust aerosol was generated using a dust generator and compressed air. Rats were restrained in glass tubes and their snouts projected into the inhalation system (nose-only exposure). Clinical observations and body weights were recorded for each animal prior to exposure and during the 14-day observation period. Mortality/morbidity observations were performed twice daily (Mon–Fri) and at least once on weekends and holidays. If moribund animals were found, they would be sacrificed humanely and necropsied. Procedures addressing recognition and handling of moribund animals for this study type are defined in a standard operation procedure of the test facility (SOP-INT 7.1.06, equivalent criteria as listed above). At the end of observation, the animals were sacrificed with CO_2_-inhalation in a chamber with increasing concentration over time.

### In vitro skin and eye irritation studies

#### EpiDerm™ skin irritation test

The *in vitro* EpiDerm™ skin irritation test was conducted in compliance with OECD Guidelines 439, updated and adopted 25 July 2015, and with EU Commission Regulation 640/2012 (2012). The study was performed in accordance with OECD Principles of Good Laboratory Practice (ENV/MC/CHEM(98)17). These principles meet the US EPA GLP standards 40 CFR Part 160 (FIFRA) and Part 792 (TSCA).

Approximately 25 μL bulk volume (about 18 mg) undiluted test article SD was applied to the surface of the EpiDerm™ tissue after application of a small amount (25 μL) of sterile PBS. Control tissues were treated with sterile PBS (negative control) or 5% SDS (positive control). Killed tissues were treated with test article or PBS to determine the direct reduction of 3-(4,5-dimethylthiazol-2-yl)-2,5-diphenyltetrazolium bromide (MTT) by the test article.

#### EpiOcular™ eye irritation test

The *in vitro* EpiOcular™ eye irritation test was conducted in compliance with OECD Guideline 492, updated and adopted 25 June 2018. The study was performed in accordance with OECD Principles of Good Laboratory Practice (ENV/MC/CHEM(98)17). These principles meet the US EPA GLP standards 40 CFR Part 160 (FIFRA) and Part 792 (TSCA).

An approximate bulk volume of 50 μL (about 26 mg) undiluted test article SD was applied to the surface of the EpiOcular™ tissue after application of a small amount (20 μL) of PBS. Control tissues were treated with sterile deionized water (negative control) or 50 μl methyl acetate (positive control). Killed tissues were treated with test article or PBS to determine the direct reduction of MTT by the test article.

## Results

### Test article chemical and biological characterization

The liquid mannanase 19287 test articles RB2 and RB5 passed all specifications, contained >70,000 TMU/kg of mannanase activity and demonstrated equivalent chemical and microbiological characteristics, including the absence of antimicrobial activities and absence of the production organism (APO). The spray-dried test article SD also passed chemical and microbiological requirements, seen in Table 1. Since test article SD is produced from RB5, the determination of antimicrobial activity and APO was not repeated. The results confirm that the test articles comply with the microbial and chemical specifications established for enzyme preparations used in food processing, as published in the FCC, Eleventh Edition, 2018 [24], and by the Joint FAO/WHO Expert Committee on Food Additives (JECFA, 2006) [25]. The results also confirm the test articles to be free of antimicrobial activity and mycotoxins and pass specifications for lead content.

**Table 1:** Characteristics of test articles RB2, RB5 and SD used in this study.

	RB2	RB5	SD
Enzyme activity [TMU/g]	89550	90890	232770
Total Organic Solids (TOS), calc. [g/100g]	23.1	23.3	67.5
Pb [≤ 5 mg/kg]	pass	pass	pass
Salmonella, absent in 25 g	pass	pass	pass
Coliforms [≤ 30 CFU/g]	pass	pass	pass
E.coli, absent in 25 g	pass	pass	pass
Antimicrobial activity	absent	absent	see RB5
Production strain	absent	absent	see RB5
Mycotoxins	absent	absent	see RB5

### Genotoxicity studies

#### Bacterial reverse mutation assay

The purpose of this study was to investigate the potential of test article RB5 to induce reverse mutations in two independent *Salmonella typhimurium* and *Escherichia coli* reverse mutation assays in the presence and absence of an exogenous metabolic activation system. No precipitation of the test article was observed up to the highest concentration; no increase in the number of revertants were observed at any concentration of endo-1,4-β-mannanase.

The reference mutagens induced an increase in reversion rate, comparable to historical data obtained at the test site.

These results indicate test article RB5 was negative for the ability to induce reverse mutations at selected loci of *S*. *typhimurium* and *E*. *coli* strains in the presence and absence of an exogenous metabolic activation system.

#### *In vitro* micronucleus test in Human Peripheral Blood Lymphocytes (HPBL)

The purpose of this study was to evaluate the potential of test article RB5 to induce micronuclei in human lymphocytes in the presence and absence of an exogenous metabolic activation system.

PHA-stimulated human lymphocytes were exposed to the diluted test article RB5 in two pulse (4 h exposure followed by 16 h of recovery) exposure and one continuous (20 h) exposure experiment, respectively.

The pulse experiments were performed in the presence or absence of S9 metabolic activation system; cells were exposed to 250 μg/mL up to 2000 μg/mL of the active substance. During the continuous exposure experiment, the cells were exposed to 250 μg/mL up to 2000 μg/mL of the active substance in the absence of the S9 mix.

In the absence or presence of S9 mix, no cytotoxicity was detected up to the highest concentration of the test article after either pulse or continuous exposure. Additionally, no induction of micronuclei was observed up to the highest concentration of the test article. The presence of mutagens (MMC, Decolcine or CPA) significantly increased cytostasis (as the measurement of cytotoxicity) and the appearance of micronuclei. These results demonstrate that β-mannanase 19287 did not induce micronuclei. The enzyme is therefore considered non-mutagenic in the *in vitro* micronucleus test in human lymphocytes, when tested up to 2000 μg/mL.

### Subchronic toxicity study—ninety-day repeated dose oral toxicity in rats

Test article RB2 was well tolerated by Wistar rats when administered in rat feed for 3 months, containing 0, 6500, 21700, or 65000 ppm test article. Table 2 lists the observed mean daily feed intake for each treatment group and gender.

**Table 2:** Treatment groups and mean daily intakes of the test articles in the ninety-day oral toxicity study in rats.

	Concentration of test article in rat feed [ppm]	Mean daily intake test article [mg/kg/day]		Concentration of enzyme [ppm, via % TOS]	Mean daily intake enzyme (ppm, via % TOS) [mg/kg/day]		Number of animals	
Group #		Male	Female		Male	Female	Male	Female
1—Control	0	0	0	0	0	0	10	10
2—Test article, low dose	6500	453	500	1500	105	116	10	10
3—Test article, mid dose	21700	1524	1645	5000	352	380	10	10
4—Test article, high dose	65000	4836	5617	15000	1117	1297	10	10

The mannanase enzyme activity for test article RB2 was measured at 89550 TMU/g, so the rat feed incorporated the following activities:

- Group 2: 6500 mg/kg x 89.55 TMU/mg = 5.82 x 10^5^ TMU/kg
- Group 3: 21700 mg/kg x 89.55 TMU/mg = 1.94 x 10^6^ TMU/kg
- Group 4: 65000 mg/kg x 89.55 TMU/mg = 5.82 x 10^6^ TMU/kg

There was no evidence of test article related toxicity, as judged by clinical observation, body weight, food consumption, clinical pathology, ophthalmology, necropsy and histopathology data evaluation. Summary data on mean body weights, hematology and serum chemistry are presented in Tables 3–5.

**Table 3:** Mean body weights (in grams) of Wistar rats during the 90-day study.

**Males**														
Group	day 0	day 7	day 14	day 21	day 28	day 35	day 42	day 49	day 56	day 63	day 70	day 77	day 84	day 91
1/M														
Means	147.7 n	190.8 n	231.8 n	261.2 n	288.2 n	309.1 n	327.4 n	340.8 n	353.1 n	363.2 n	371.3 n	380.4 n	387.0 n	386.3 n
Sdevs	7.9	11.7	14.6	19.4	21.8	25.5	26.5	28	29.4	32.2	31.8	36.6	35.6	34.5
2/M														
Means	148.0	190.7	230.0	258.0	284.2	308.4	327	341.9	352.4	364.4	372.3	376.5	389.3	393.1
Sdevs	6.8	8.1	11.9	15.2	17.2	22.4	25.6	28.5	30.4	33.6	35.3	35.1	37.1	38.5
3/M														
Means	147.5	191.2	234.6	267.8	295.8	321.6	339.5	354.3	366.6	378.1	387.6	397.7	403.1	406.3
Sdevs	6.8	9.5	12.8	14.2	17.5	20.1	22.1	23.8	26.2	28.2	29.1	31	32.7	32.3
4/M														
Means	146.3	189.2	230.0	263.1	287.6	308.5	325.3	336.5	348.1	358.4	365.9	375.1	383.7	386.9
Sdevs	7.1	9.9	12.3	14.2	15.8	17.9	19.7	20.7	22.3	23.3	26.4	29.4	30.8	31.3
**Females**														
Group	day 0	day 7	day 14	day 21	day 28	day 35	day 42	day 49	day 56	day 63	day 70	day 77	day 84	day 91
1/F														
Means	118.9 n	138.4 n	155.9 n	171.0 n	183.5 n	192.4 n	200.4 n	206.7 n	211.6 n	214.4 n	216.2 n	220.4 n	223.4 n	220.3 n
Sdevs	6	5.7	9.3	10	9.2	9.4	11.5	11.8	8.9	8.8	12.2	11.2	9.9	8.3
2/F														
Means	118.4	141.9	160.7	176.6	186.1	198.8	205.2	212.9	217.3	222.4	225.1	228.4	232.2	235.6*
Sdevs	6.5	7.5	8.9	11.3	12.5	11.1	10.7	11.9	12.3	11.6	12.8	13.8	16.7	15.3
3/F														
Means	119.8	139.4	158.7	173.5	183.8	196.1	203.3	209.9	214.4	218.8	221.2	225.4	229.3	229.3
Sdevs	5.3	5.6	6.7	8	5.6	7	7.6	7.4	5.3	6.8	8.1	8.4	5.9	6.5
4/F														
Means	119	138.1	154.1	169.1	179.8	190.6	196.6	201.5	208.7	211.7	213	217.9	221.6	223.4
Sdevs	5.2	7	9.7	9.3	8.7	11.8	12.2	9.8	11.3	11.7	15	12.2	11.5	13.7

Each group consisted of 10 animals per sex and are identified as follows: 1/M, 1/F = 0 mg/kg/day, 2/M = 105 mg/kg/day, 2/F = 116 mg/kg/day, 3/M = 352 mg/kg/day, 3/F = 380 mg/kg/day, 4/M = 1117 mg/kg/day, and 4/F = 1297 mg/kg/day

Statistic Profile = Dunnett test (two-sided)

* p< = 0.05

** p < = 0.01

n = DUNNETT

**Table 4:** Hematology summary for the 90-day study.

	Males								Females							
	Group 1/M		Group 2/M		Group 3/M		Group 4/M		Group 1/F		Group 2/F		Group 3/F		Group 4/F	
	Means	Sdevs	Means	Sdevs	Means	Sdevs	Means	Sdevs	Means	Sdevs	Means	Sdevs	Means	Sdevs	Means	Sdevs
WBC	5.59 k	1.12	5.43	1.35	5.77	1.32	5.00	1.28	2.99 k	0.60	2.80	0.81	2.85	0.69	2.35	0.75
RBC	8.57 k	0.27	8.69	0.40	8.49	0.32	8.54	0.29	7.72 k	0.36	7.41	0.29	7.76	0.36	7.63	0.27
HGB	8.4 k	0.3	9.1	0.2	8.9	0.3	8.8	0.2	8.4 k	0.3	8.2	0.2	8.5	0.2	8.4	0.4
HCT	0.396 k	0.014	0.430	0.009	0.427	0.012	0.421	0.012	0.396 k	0.014	0.387	0.009	0.399	0.012	0.394	0.020
MCV	49.6 k	1.3	49.5	2.0	50.3	1.7	49.4	1.6	51.3 k	0.8	52.3	1.4	51.6	1.8	51.7	1.4
MCH	1.04 k	0.03	1.05	0.04	1.06	0.04	1.04	0.04	1.09	K	0.03	1.11	0.04	1.09	0.05	1.10
MCHC	21.06 k	0.19	21.19	0.23	20.96	0.27	21.03	0.39	21.26 k	0.29	21.18	0.31	21.20	0.53	21.19	0.39
PLT	701 k	72	696	82	696	40	670	62	658 k	78	669	40	686	67	701	78
%NEUT	20.1 k	6.7	15.9	4.3	18.3	2.5	18.0	4.4	6.2	22.4	4.7	20.8	6.4	20.7	9.5	
%LYMPH	75.6 k	7.1	80.1	4.8	77.1	2.8	77.4	4.8	79.7 k	6.7	72.8	5.6	74.8	7.0	73.7	8.7
%MONO	1.8 k	0.5	1.7	0.4	1.8	0.6	2.1	0.7	1.5 k	0.5	1.9	0.6	1.4	0.6	1.9	0.8
%EOS	2.1 k	0.5	1.7	0.5	2.2	0.6	2.0	0.5	2.4 k	0.7	2.3	0.9	2.5	1.0	3.2	1.0
%BASO	0.3 k	0.1	0.3	0.2	0.3	0.2	0.2	0.1	0.3 k	0.1	0.2	0.2	0.2	0.1	0.3	0.2
%LUC	0.2 k	0.2	0.3	0.1	0.3	0.1	0.3	0.1	0.2 k	0.1	0.3	0.2	0.2	0.1	0.2	0.1
#NEUT	1.12 k	0.41	0.84	0.20	1.04	0.19	0.87	0.21	0.46 k	0.15	0.62	0.16	0.58	0.18	0.46	0.20
#LYMPH	4.22 k	0.93	4.38	1.27	4.46	1.11	3.90	1.09	2.40 k	0.63	2.06	0.68	2.15	0.63	1.76	0.69
#MONO	0.10 k	0.04	0.09	0.02	0.10	0.05	0.10	0.05	0.04 k	0.02	0.05	0.01	0.04	0.02	0.04	0.02
#EOS	0.12 k	0.04	0.09	0.02	0.12	0.03	0.10	0.03	0.07 k	0.02	0.06	0.03	0.07	0.02	0.08	0.04
#BASO	0.02 k	0.01	0.02	0.01	0.02	0.01	0.01	0.00	0.01 k	0.00	0.00	0.01	0.01	0.01	0.00	0.01
#LUC	0.02 k	0.01	0.02	0.01	0.02	0.01	0.01	0.01	0.01 k	0.01	0.01	0.00	0.00	0.01	0.00	0.01
#RETIC	138.7 v	18.6	134.8	25.0	162.7**	19.9	156.1	20.7	138.9 k	32.6	171.0	38.3	156.0	32.1	164.3	32.4

Each group consisted of 10 animals per sex and are identified as follows: 1/M, 1/F = 0 mg/kg/day, 2/M = 105 mg/kg/day, 2/F = 116 mg/kg/day, 3/M = 352 mg/kg/day, 3/F = 380 mg/kg/day, 4/M = 1117 mg/kg/day, and 4/F = 1297 mg/kg/day

WBC = white blood cells (x10e3/μL), RBC = red blood cell (x10e6/μL), HGB = hemoglobin (g/dL), HCT = hematocrit (%), #LYMPH = lymphocytes (x10e3/μL), %LYMPH = lymphocytes % (%), MCV = mean corpuscular volume (fL), MCHC = mean corpuscular hemoglobin concentration (g/dL), MCH = mean corpuscular hemoglobin (pg), #MONO = absolute monocytes (x10e3/μL), %MONO = monocytes % (%), #NEUT = absolute neutrophil (x10e3/μL), %NEUT = neutrophil % (%), PLT = platelets (x10e3/μL), #BASO = absolute basophil (x10e3/μL), %BASO = basophil % (%), #EOS = absolute eosinophil (x10e3/μL), %EOS = eosinophil % (%); #LUC = absolute large unstained cells (x10e3/μL), %LUC = large unstained cells % (%), and #RETIC = absolute reticulocyte (x10e9/L).

Statistic Profile = Kruskal-Wallis + Wilcoxon test (two-sided)

* p< = 0.05

** p < = 0.01, X = Group excluded from statistics; k = KRUSKALL-WALLIS; v = KRUSKALL-WALLIS-WILCOX

**Table 5:** Serum chemistry summary for the 90-day study.

	Males								Females							
	Group 1/M		Group 2/M		Group 3/M		Group 4/M		Group 1/F		Group 2/F		Group 3/F		Group 4/F	
	Means	Sdevs	Means	Sdevs	Means	Sdevs	Means	Sdevs	Means	Sdevs	Means	Sdevs	Means	Sdevs	Means	Sdevs
***Enzymes***																
ALT	0.64 v	0.09	0.76*	0.10	0.64	0.07	0.79	0.31	0.57 v	0.08	0.46**	0.08	0.60	0.09	0.52	0.10
AST	1.54 k	0.19	1.85	0.37	1.70	0.30	1.79	0.28	1.73 v	0.17	1.48*	0.25	1.55*	0.25	1.49*	0.20
ALP	1.14 k	0.26	1.17	0.24	1.13	0.17	1.08	0.17	0.43 k	0.09	0.60	0.15	0.50	0.16	0.50	0.14
GGT_C	25 NA	0	25	0	25	0	25	0	25 NA	0	25	0	25	0	25	0
***Substrates***																
UREA	5.56 k	0.44	5.77	0.90	5.24	0.60	5.54	0.67	6.69 k	0.69	6.45	1.06	6.53	0.86	6.66	1.00
CREA	26.8 k	3.6	28.9	4.7	27.2	1.6	28.4	2.4	34.6 k	3.4	33.7	3.4	32.5	4.4	34.7	5.8
GLUC	6.95 k	0.58	7.38	0.96	7.43	0.91	7.45	0.88	5.70 k	0.82	5.87	0.70	5.88	0.76	5.62	0.54
TBIL	1.23 k	0.22	1.51	0.39	1.20	0.23	1.14	0.26	1.58 k	0.36	1.54	0.23	1.58	0.43	1.50	0.28
TBA	8.2 k	4.4	12.1	7.2	11.6	10.7	9.1	6.6	22.3 k	12.3	15.1	8.1	25.0	7.6	19.8	13.0
TPROT	63.10 k	2.42	62.28	1.43	63.87	2.02	63.15	1.69	65.45 k	2.27	64.67	3.47	66.75	3.16	65.00	2.28
ALB	36.16 k	1.41	35.67	0.97	36.26	0.75	35.83	0.63	38.19 k	1.38	38.18	2.10	39.16	2.61	38.99	1.45
GLOB	26.93 k	1.61	26.61	1.05	27.61	1.46	27.31	1.69	27.26 k	1.41	26.49	2.19	27.59	1.34	26.01	1.50
CHOL	1.41 k	0.30	1.56	0.30	1.56	0.28	1.60	0.29	1.34 k	0.28	1.23	0.17	1.25	0.25	1.09	0.30
HDL-CHOL	1.02 k	0.24	1.15	0.25	1.14	0.20	1.15	0.20	1.17 k	0.25	1.09	0.12	1.11	0.22	0.98	0.24
LDL-CHOL	0.17 k	0.05	0.21	0.07	0.20	0.07	0.22	0.09	0.11 k	0.01	0.10	0.01	0.10	0.01	0.10	0.01
TRIG	1.10 k	0.36	0.98	0.35	1.16	0.34	1.06	0.27	0.57 k	0.11	0.50	0.18	0.54	0.17	0.51	0.24
***Electrolytes and minerals***																
Na	142.4 k	0.8	141.4	1.2	142.5	0.7	142.3	1.2	141.7 k	1.9	141.2	1.4	142.5	1.9	141.8	1.1
K	4.51 k	0.21	4.62	0.38	4.56	0.23	4.50	0.20	4.11 k	0.21	3.91	0.17	3.84	0.23	3.91	0.21
Cl	98.7 k	1.1	98.2	1.2	99.2	1.0	98.8	1.5	100.2 k	1.5	100.0	1.3	100.0	2.2	99.9	1.6
PHOS	1.59 k	0.17	1.75	0.16	1.58	0.18	1.56	0.21	1.11 k	0.11	1.06	0.26	1.10	0.16	1.00	0.09
Ca	2.52 k	0.04	2.54	0.05	2.56	0.04	2.52	0.06	2.55 k	0.06	2.50	0.07	2.53	0.06	2.53	0.04

Each group consisted of 10 animals per sex and are identified as follows: 1/M, 1/F = 0 mg/kg/day, 2/M = 105 mg/kg/day, 2/F = 116 mg/kg/day, 3/M = 352 mg/kg/day, 3/F = 380 mg/kg/day, 4/M = 1117 mg/kg/day, and 4/F = 1297 mg/kg/day

Enzymes: ALT = alanine aminotransferase (μkat/L/L), AST = aspartate aminotransferase (μkat/L), ALP = alkaline phosphatase (μkat/L), GGT_C = serum-γ-glutamyltransferase (nkat/L).

Substrates: UREA = urea (mmol/L), CREA = creatinine (μmol/L), GLUC = glucose (mmol/L), TBIL = total bilirubin (μmol/L), TBA = bile acids (μmol/L), TPROT = (g/L), ALB = albumin (g/L), GLOB = globulin (g/L), CHOL = cholesterol (mmol/L), HDL-CHOL = HDL-cholesterol (mmol/L), LDL-CHOL = LDL-cholesterol (mmol/L), TRIG = triglycerides (mmol/L).

Electrolytes and minerals: Na = sodium (mmol/L), K = potassium (mmol/L), Cl = chloride (mmol/L), PHOS = inorganic phosphate (mmol/L), Ca = calcium (mmol/L).

Statistic Profile = Wilcoxon test (one-sided+), Kruskal-Wallis + Wilcoxon test (two-sided)

* p< = 0.05

** p < = 0.01.

v = KRUSKALL-WALLIS-WILCOX; k = KRUSKALL-WALLIS; NA = No Test Applicable.

No deaths or overt signs of toxicity were observed during the course of the study. No clinical findings were observed for male and female animals of any test group. No test substance-related changes in body weight parameters were observed in male and female animals of any test group (Table 3). Mean body weight and body weight change values were significantly higher in female animals of test group 2/F (6500 ppm of test article RB2, i.e. 116 mg TOS/kg bw/day) on study day 91. These changes were considered to be incidental because of a missing dose-response relationship.

For hematology values (Table 4), no treatment related changes were observed. At the end of the administration period, absolute reticulocyte counts were significantly increased in males of test group 3/M (21700 ppm of test article RB2, i.e. 352 mg TOS/kg bw/day), but this change was not dose dependent. Therefore, it was regarded as incidental and not treatment related.

For serum chemistry values (Table 5), no treatment related changes among clinical chemistry parameters were observed. At the end of the administration period, alanine aminotransferase (ALT) activities were significantly increased in males of test groups 2/M (6500 ppm, i.e. 105 mg/kg/day), whereas in females of the same test group ALT activities were significantly decreased. In females of test groups 2/F, 3/F and 4/F (6500, 21700 and 65000 ppm of test article RB2, i.e. 116, 380 and 1297 mg TOS/kg bw/day, respectively) aspartate aminotransferase (AST) activities were significantly lower compared to controls. However, all mentioned alterations were not dose dependent, and, therefore, they were regarded as incidental and not treatment related.

No differences were observed in clinical pathology parameters, nor any macroscopic or microscopic alterations in tissues examined post-mortem. No adverse findings were noted at the highest dose tested. In conclusion, NOAEL may be >1117 mg TOS/kg bw/day (in male rats), >1297 mg TOS/kg bw/day (in female rats), or >4.33–5.03 x 10^5^ TMU/kg bw/day (for male and female rats, respectively).

### Acute inhalation toxicity

In order to evaluate the acute inhalation toxicity of the active substance endo-1,4-β-mannanase as a dust, the test article SD was first analyzed for the particle size distribution of the sample. The results demonstrated that the dust particles are within the respirable range per OECD Guideline 403. Subsequently, the test animals were exposed to the test article dust for four hours at the rate of 5.09 mg test article SD/L air. The animals were housed for 14 days after inhalation testing and checked for clinical observations, body weight and mortality rate.

No mortality was observed after testing at the maximum rate > 5.1 mg/L air. Clinical observations showed intermittent respiration in the animals up to day 5; no clinical signs and findings were observed from day 6 to day 14. Mean body weights decreased on the first day post-inhalation; male rats increased their weights starting on day 2, female rats after day 3 post-testing. Gross pathology examination showed no signs of pathological abnormalities during necropsy after the animals were euthanized on day 14.

Based on the results of the study, the LC50 is > 5.1 mg/L air in male and female rats after the four-hour exposure to dust aerosol of the active substance endo-1,4-β-mannanase (mannanase 19287).

### In vitro skin and eye irritation studies

#### EpiDerm™ skin irritation

The potential of the active substance endo-1,4-β-mannanase (mannanase 19287) to cause skin irritation was evaluated by exposing the *in vitro* test system EpiDerm™ to the test article SD. A single dose SD was applied to the three-dimensional human epidermis model EpiDerm for 1 h. Subsequently, the epidermal constructs were maintained for 42 h by incubation with cell culture medium. Irritation was determined as tissue destruction by measuring the metabolic activity of the tissue using a colorimetric test. Since the test article independently shows a colorimetric reaction with the reagent, EpiDerm™ tissue samples were killed by freezing at –20°C and served as non-viable controls. Viable tissues and non-viable control tissues were exposed to PBS (non-cytotoxic, negative control, NC), 5% SDS (cytotoxic, positive control; PC) and the test article. Substances that demonstrate tissue viability of > 50% of the negative control (PBS) are considered non-irritant in the test system.

Non-viable tissues demonstrated a metabolic activity of 0.2% of the PBS negative control; this metabolic activity was used to adjust the outcome of the test articles on the EpiDerm™ viability. The single dose exposure of the endo-1,4-β-mannanase test article resulted in a mean viability of 98.5% compared to the PBS NC; therefore, mannanase 19287 does not show a skin irritation potential. In contrast, the 5% SDS PC resulted in < 3% tissue viability compared to the NC.

#### EpiOcular™ eye irritation

The potential of the active substance endo-1,4-β-mannanase (mannanase 19287) to cause eye irritation was evaluated by exposing the *in vitro* test system EpiOcular™ to the test article SD. A single dose of test article SD was applied to the three-dimensional human cornea model EpiOcular for 6 h. Subsequently, the cornea constructs were maintained for 18 h by incubation with cell culture medium. Irritation was determined as tissue destruction by measuring the metabolic activity of the corneal tissue using a colorimetric test. Since the test article independently shows a colorimetric reaction with the reagent, non-viable tissue was included as negative controls.

After exposure of the tissues to PBS (negative control), the test article, and the test article to non-viable tissue, tissue destruction was determined by comparison of the metabolic activity to the metabolic activity of the negative control. When corrected by the activity of the killed tissue, the test article exposure resulted in 88.6% viability of the corneal tissue; in contrast, exposure to the positive control (neat methyl-acetate) resulted in 13.6% viability.

Based on the results, the active substance mannanase 19287 does not show an eye irritation potential in EpiOcular™ test system.

## Discussion

The fungal microorganism *T*. *thermophilus* DSM 33149 is the production strain for the manufacture of mannanase 19287, an endo-1,4-β-mannanase (E.C. 3.2.1.78). The resulting enzyme preparation was evaluated using liquid test articles RB2 and RB5, as well as the spray-dried material derived from RB5.

Assessment of the safety of the production strain was conducted in compliance with the safety guidelines established for feed additives by Pariza and Cook [28], which references earlier work that defines a non-toxigenic organism as “one that does not produce injurious substances at levels that are detectable or demonstrably harmful under ordinary conditions of use or exposure.” [29], as well as with various safety considerations for human food additives established by the International Food Biotechnology, OECD, Pariza and Foster, and Pariza and Johnson [29–31]. In addition, the potential for the enzyme preparation to induce allergenicity or for the production strain to produce mycotoxins or antimicrobial substances [32], respectively, was also considered. As discussed below, the *T*. *thermophilus* production strain DSM 33149 fulfills the criteria of a nontoxigenic and nonpathogenic microorganism.

A review of the literature shows that *T*. *thermophilus* is generally considered unlikely to be more than a rare opportunistic pathogen in immunocompromised or post-traumatic or post-surgery patients. A Letter to the Editor of Clinical Microbiology and Infection describes five clinical human infections of *T*. *thermophilus* and compares the outcomes with seven published reports ranging from 1990 to 2016 [33]. Pulmonary or disseminated infections were observed exclusively in five immunocompromised patients. Four of the 12 cases ended with the death of the patient, three immunocompromised individuals and one recipient of repeated aortic grafts from human donors within four months.

The wild-type *T*. *thermophilus* C1 strain has been developed into the non-pathogenic and non-toxigenic strain HC [16]; host- and production strains derived from *T*. *thermophilus* HC have been used in industrial settings for over 20 years. See Fig 1 above for the strain lineage from the native C1 strain to strain HC and the mannanase 19287 production strain DSM 33149.

Dyadic International (USA), Inc., the manufacturer of a cellulase enzyme preparation derived from a genetically modified *T*. *thermophilus* HC strain (still classified as *Myceliophthora thermophila*), determined that the preparation is GRAS for the intended use during beer, wine and juice production for human consumption and notified FDA/CFSAN of their GRAS determination [17]. The notification included summaries of unpublished toxicity, toxigenicity and pathogenicity studies. FDA issued a No Questions letter for GRAS Notification GRN 292 to Dyadic International (USA), Inc. [18].

In addition, a laccase from *M*. *thermophila* expressed in *Aspergillus oryzae* was the subject of a published toxicological study [34] and separately evaluated by JECFA [35]. No peer-reviewed safety evaluations of enzyme preparations produced in *T*. *thermophilus* have been identified in the literature.

Oral allergenicity is not a concern for enzyme animal feed additives; however, if inhaled, all enzyme preparations have the potential to elicit sensitization in workers exposed during the manufacturing or packaging. Several safe handling guidelines are available to minimize the risk of exposure [36–38].

To evaluate the safety of production strain DSM 33149 for the heterologous expression of an animal feed grade enzyme, the test articles were produced using a process representative of the manufacturing process at commercial scale and were used in the toxicity studies described in this publication. No genotoxicity was reported in the mutagenicity assay or the micronucleus assay in human lymphocytes. Further, there was no *in vivo* toxicity reported in the acute inhalation and subchronic oral toxicity studies. In addition, no irritation was observed in *in vitro* dermal and eye irritation safety studies.

The NOAEL from the subchronic toxicity study together with the use level of the enzyme in feed applications can be used to calculate the level of safety for the enzyme preparation. The safety margin is calculated by dividing the NOAEL from the 90-day oral toxicity study by the estimated daily intake (EDI) for broilers at the target rate of enzyme utilization (800 TMU/kg feed). For example, a safety factor of 100 to 1 has been codified in the US in 21CFR170.22 when using animal toxicity data for human food additives. This safety factor is also listed in several publications and regulatory guidances, e.g. [28, 29, 31, 39]. A similar 100-fold safety factor can be considered for the comparison of toxicity data in rats to a safe consumption rate in broiler chicken.

The highest dose level of test article RB2 (89.55 TMU/mg) fed to Group 3 rats in the 90-day oral subchronic toxicity study was 65,000 mg/kg feed. The average daily test article intake for Group 3 animals (65,000 mg/kg feed) was 4836 mg test article/kg body weight/day (mg/kg bw/d) for male rats and 5617 mg/kg bw/d for female rats (see Table 2). Based on the enzyme activity of the test article (89.55 TMU/mg), male and female rats ingested:

- Male rats: 4836 mg test article/kg bw/d x 89.55 TMU/mg = 4.33 x10^5^ TMU/kg bw/d
- Female rats: 5617 mg test article/kg bw/d x 89.55 TMU/mg = 5.03 x10^5^ TMU/kg bw/d

Since none of the experimental groups showed adverse effects, the NOAEL (TMU) values for the endo-1,4-β-Mannanase during the 90-day oral toxicity study range from 4.33–5.03 x 10^5^ TMU/kg bw/d.

The original Pariza publication examining the safety of food enzyme preparations from non-toxigenic organisms recommends the use of TOS when determining a reliable estimate of enzyme use and consumption [29]. TOS is defined as the sum of the organic compounds, excluding diluents, contained in the final enzyme preparation (TOS (%) = 100 - % ash—% water—% diluent).

Pariza and co-workers have updated the safety evaluation of enzymes to include preparations derived from recombinant production strains [31] and subsequently expanded their approach to enzymes used in animal feed [28].

Broilers will be exposed to the enzyme immediately after hatching and will consume varying amounts of food per body weight, starting initially at 22% and slowly decreasing to just under 8%. All broiler data listed below are obtained from the 2019 Ross-708 Performance Objectives [40] as hatched.

On day 42, the average broiler weighs 2.78 kg and consumes 204 g of feed; this is the highest estimated daily intake or EDI_max_ (feed) is 72.86 g/kg bw/d. Based on the cumulative feed intake of 4430 g by day 42, the average estimated daily intake or EDI_avg_ (feed) equals 105.5 g/day (4430 g/ 42 days) and the average intake per kg body weight is 37.95 g/kg bw/d. Both EDI_max_ (feed) and EDI_avg_ (feed) were used to calculate safety factors for the enzyme.

The % TOS for the endo-1,4-β-Mannanase preparation used in the 90-day oral toxicity study has been calculated as 23.1% or 0.231 g TOS/g enzyme. The direct % TOS determination of the dry enzyme preparation (8000 TMU/kg) used for broiler feed is not feasible, since the carrier itself has a high TOS content. The TOS value for the endo-1,4-β-Mannanase preparation used in the broiler study was estimated at 2.64 g TOS/1000 kg feed; this value is based on the % TOS of the enzyme intermediate product (0.675 g TOS/g) mixed with the carrier during the formulation of the powdered Natupulse^®^ TS product.

In the 90-day oral toxicity rat study, the mean daily intake of test article RB2 (% TOS = 23.1% or 0.231 mg TOS / mg Test Article) for Group 3 animals (65000 ppm) was 4836 mg/kg bw/d (male rats) and 5617 mg/kg bw/d for female rats, respectively. These daily intakes result in the following NOAEL values:

$$\begin{array}{c} •\;NOAEL\;male=4836\;mg/kg\;bw/d\;x\;0.231\;mg\;TOS/mg\;test\;article=\\ 1117\;mg\;TOS/kg\;bw/d \end{array}$$

$$\begin{array}{c} •\;NOAEL\;female=5617\;mg/kg\;bw/d\;x\;0.231\;mg\;TOS/mg\;test\;article=\\ 1298\;mg\;TOS/kg\;bw/d \end{array}$$

### Safety factors (maximum intake)

The maximal broiler estimated daily intake, EDI_max_ (feed) of 72.86 g feed/kg bw/d, can be converted to EDI_max_ (TOS) for the target rate of 800 TMU/kg broiler feed as follows:

$$\begin{array}{c} •\;EDI_{max}=72.86\;g\;feed/kg\;bw/d\;x\;2.64\;x\;10^{‐6}\;g\;TOS/g\;feed=\\ 1.92\;x\;10^{‐4}\;g\;TOS/kg\;bw/d=\\ 0.192\;mg\;TOS/kg\;bw/d \end{array}$$

Safety factors are calculated as NOAEL ÷ EDI_max_:

- Safety factor (male) = 1117 mg TOS/kg bw/d ÷ 0.192 mg TOS/kg bw/d = 5818
- Safety factor (female) = 1298 mg TOS/kg bw/d ÷ 0.192 mg TOS/kg bw/d = 6760

### Safety factors (average intake)

The average broiler feed consumption over the 42-day lifespan or EDI_avg_ (feed) of 37.95 g/kg bw/d can be converted to the EDI_avg_ (TOS) at the target rate of 800 TMU/kg broiler feed:

$$\begin{array}{c} •\;EDI_{avg}\;\left(TOS\right)=37.95\;g/kg\;bw/d\;x\;2.64\;x\;10^{‐6}\;g\;TOS/g\;feed=\\ 1.0\;x\;10^{‐4}\;g\;TOS/kg\;bw/day=\\ 0.1\;mg\;TOS/kg\;bw/d. \end{array}$$

This results in the following safety factors using average intake:

- Safety factor (male) = 1117 mg TOS/kg bw/d ÷ 0.1 mg TOS/kg bw/d = 11170
- Safety factor (female) = 1298 mg TOS/kg bw/d ÷ 0.1 mg TOS/kg bw/d = 12980

Table 6 summarizes the safety factors calculated for broiler feed consumption over 42 days and the maximal feed consumption on day 42. Applying the codified 100-fold safety factor for animal toxicity data and human food additives (see above) to our current interspecies extrapolation from rat to chicken, the safety factors are in 50- to 100-fold excess of the regulatory 100 to 1 requirement per 21 CFR 170.22.

**Table 6:** Safety factors for endo-1,4-β-Mannanase enzyme preparation.

	EDI_max_ [mg TOS/kg bw/d]	EDI_avg_ [mg TOS/kg bw/d]	NOAEL [mg TOS/kg bw/d]	Safety Factor
Maximal consumption	0.192		1117 (male)	5818
			1298 (female)	6760
Average consumption		0.1	1117 (male)	11170
			1298 (female)	12980

Safety factors are calculated as the ratio of NOAELs of the 90-day rat study to the EDIavg (over 42 days) or the EDImax (on day 42) broiler feed consumption at the target rate of 800 TMU/kg feed.

While not within the scope of this manuscript, please note that studies performed in target animals confirmed the previously reported effects of β-mannanase addition to animal feed containing significant quantities of mannans. Mannans and other SNSPs affect the viscosity of digesta in farm animals and addition of β-mannanase preparations ameliorate these effects [5, 6]. Our broiler studies demonstrated that addition of mannanase 19287 resulted in dose-dependent reduction of digesta viscosities when compared to mannan-containing diets without mannanase 19287 present (not shown).

## Conclusion

The safety evaluation of the test article clearly demonstrates that the mannanase 19287 enzyme preparation here in question:

1. Is produced by a non-toxigenic and non-pathogenic production strain (DSM 33149) generated from a parental *T*. *thermophilus* strain which has a history of safe use.
2. Is derived from a controlled, pure fermentation process.
3. Does not induce any *in vitro* genotoxicity or any *in vivo* toxicity in rats when consumed at levels up to 5617 mg/kg bw/d, and
4. Using TOS, has a safety margin of 5818–6760 based on the NOAELs of 1117–1298 mg TOS/kg bw/d and the conservative broiler consumption estimate of EDI_max_ (TOS) = 72.86 g/kg bw/d on day 42.

We conclude that the mannanase 19287 enzyme preparation is safe for the intended use as an animal feed additive to reduce digesta viscosity. We base our conclusion on the results of the toxicology studies described in this first peer-reviewed safety evaluation for an enzyme preparation produced with *T*. *thermophilus*. In addition, the available safety record of this species and the published literature for mannanases in general support our safety conclusion.
